# A novel deletion mutation in the *BCOR* gene is associated with oculo-facio-cardio-dental syndrome: a case report

**DOI:** 10.1186/s12887-022-03148-x

**Published:** 2022-02-07

**Authors:** Qian Hu, Jingqun Mai, Qinqin Xiang, Bin Zhou, Shanling Liu, Jing Wang

**Affiliations:** 1 Laboratory of Molecular Translational Medicine, Centre for Translational Medicine, Key Laboratory of Birth Defects and Related Diseases of Women and Children (Sichuan University), Ministry of Education, Clinical Research Center for Birth Defects of Sichuan Province, West China Second University Hospital, Sichuan University, Chengdu, Sichuan 610041 P.R. China; 2 Department of Obstetrics and Gynecology, West China Second University Hospital of Sichuan University, Chengdu, 610041 WH China; 3 Key Laboratory of Birth Defects and Related Diseases of Women and Children, Ministry of Education, Sichuan University, Chengdu, 610041 WH China

**Keywords:** Oculo-facio-cardio-dental syndrome, BCOR, X-linked development disorder, Cardiac disease, Case report

## Abstract

**Background:**

Oculo-facio-cardio-dental syndrome is a rare X-linked dominant syndrome, characterized by radiculomegaly, congenital cataracts, dysmorphic facial features, and congenital heart disease. Because of the rarity, this syndrome could be misdiagnosed by the clinician, especially for the infant who may present only one to two systems involved.

**Case presentation:**

Here we report a 3-month-old female infant presenting with typical clinical manifestations of oculo-facio-cardio-dental syndrome, like ocular, facial, cardiac, and skeletal abnormalities, and the genetic analyses of the proband and her parents were provided. Genetic evaluations were completed using whole exon sequencing, which revealed a novel heterozygous mutation between exons 7 and 14 of the *BCOR* gene(OMIM:300485) in this patient but not in her parents. This mutation is likely to encode a premature stop codon producing a truncated protein. Our patient was diagnosed early enough to allow for the cardiac defects to be treated first, and she will be closely followed up to ensure that any new presentations are treated in a timeous manner.

**Conclusion:**

This patient fits the diagnostic criteria for oculo-facio-cardio-dental syndrome and is the youngest oculo-facio-cardio-dental syndrome patient ever reported, which is most important for her prognosis. In addition, this manuscript also describes a novel potenitally causative mutation for this syndrome.

## Background

Oculo-facio-cardio-dental (OFCD) syndrome, also known as Microphthalmia, syndromic 2 (OMIM 300166), is a rare pathological syndrome caused by mutations in the *BCL6* corepressor gene (*BCOR*) [1]. This syndrome usually involves multiple systems, and its typical traits include (1) eye anomalies (congenital cataracts, microphthalmia, or secondary glaucoma); (2) facial abnormalities (long narrow face, high nasal bridge, pointed nose with cartilages separated at the tip, cleft palate, or submucous cleft palate); (3) cardiac anomalies (atrial septal defect, ventricular septal defect, or floppy mitral valve); and (4) dental abnormalities (canine radiculomegaly, delayed dentition, oligodontia, persistent primary teeth, or variable root length) [2]. Hayward in 1980 first reported OFCD syndrome [3] and described radiculomegaly of the canines and congenital cataracts, and this report was supplemented with other studies conducted to explain cuspid gigantism.

OFCD syndrome has X-Linked dominant inheritance, which means that its effects are lethal in males, making all clinical patients females [1]; *BCOR* gene abnormalities account for almost all cases of this syndrome [4]. The *BCOR* gene is located in Xp11.4, where it acts as an apparent genetic regulator associated with specific cell differentiation and limb structure development [4]. Evaluations of the genetic basis of OFCD syndrome have revealed truncating, missense, frameshift, and deletion mutations in the *BCOR* gene, all of which produce premature stop codons inducing varying OFCD syndrome phenotypes [5].

Here we report the case of a 3-month-old infant presenting with OFCD syndrome, and she experienced a heterozygous loss of exons 7–14 in the *BCOR* gene whereas both her parents presented with a normal genotype. She was first admitted to the pediatric cardiovascular department for cardiac disease and then referred to the medical genetics department for genetic identification. As far as we know, she is the youngest OFCD patient ever reported, which is vital for her prognosis.

## Case presentation

### Human subjects

One female subject with OFCD syndrome and her parents underwent a complete medical genetics evaluation. All available medical records were obtained in accordance with the guidelines established by the ethics committee at West China Second University Hospital, Sichuan University.

### Molecular genetic analyses

DNA was extracted from peripheral vein blood using the DNeasy Blood and Tissue Kit (Qiagen, Hilden, Germany). The exons and splice regions from the 25129 ref gene were captured and enriched using a Nano WES capture chip (Berry gene, Hangzhou, China). A NovaSeq 6000 sequencing system (Illumina, USA) was used to sequence the captured Library (average sequencing depth > 100 x).

Sequencing data annotation and pathogenicity classification was then completed. The GATK pipeline was used for SNP / InDel calling, and the XHMM pipeline was used for CNV calling. The mutation sites were annotated according to the pathogenic mutation database (ClinVar, OMIM and HGMD), general population genome database (1000g, esp6500, ExAC, gnomAD, Berry and Kahn's Chinese population database), and hazard and conservation prediction database. The standards and guidelines for the interpretation of sequence variants recommended by the American College of medical genetics and genomics and the Association for molecular pathology were used to classify the pathogenicity of each of the variant loci.

Triplicate quantitative PCR was then performed using genomic DNA in an effort to verify the WES results using SYBR Green qPCR Master Mix (Thermo Fisher Scientific, Vilnius, Lithuania) and an Applied Biosystems 7500 Real-Time PCR Systems (Thermo Fisher Scientific, Waltham, MA, USA). The ^−2 ΔΔ^CT analysis method was used to evaluate the copy number of *BCOR* exons 8 and 14 and intron 10 in each sample using specific primer pairs (Table 1).

**Table 1:** qPCR primer pairs for Exons 8 and 14 and Intron 10 of the *BCOR* gene

Primers	Forward	Reversed
Exon 8	CTGAGGCTGGAATGAAGG	TGACAATGAACAAGGTATGC
Intron 10	CTGTGGATGTCTTGGTGAG	GATTGAGTGAGGAGGATGTC
Exon 14	ATAGGCATCGTCATCATCAT	CCGTAGGAGGTGAACAAG

### Clinical report

**Fig. 1:**
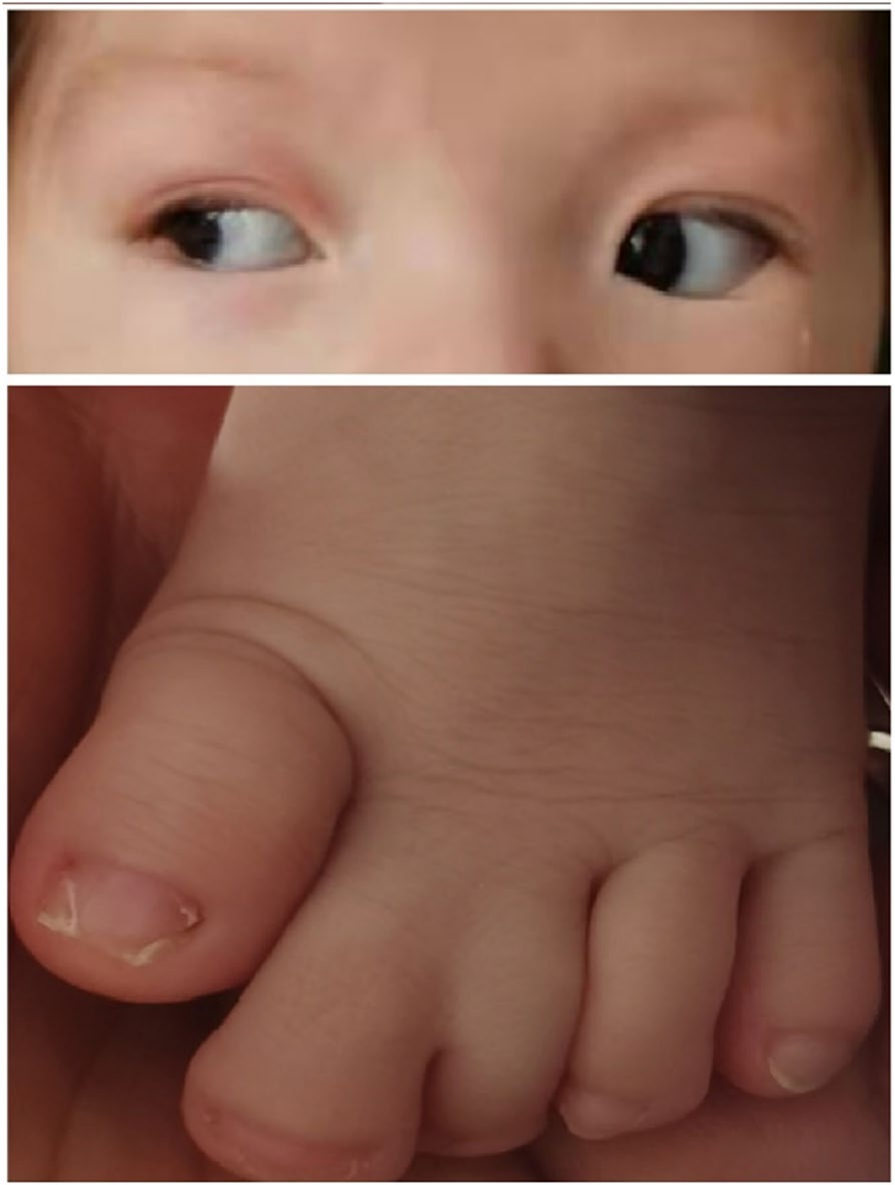
Left 2–3 toe syndactyly, true microphthalmos in the right eye and congenital cataract in the left eye

**Fig. 2:**
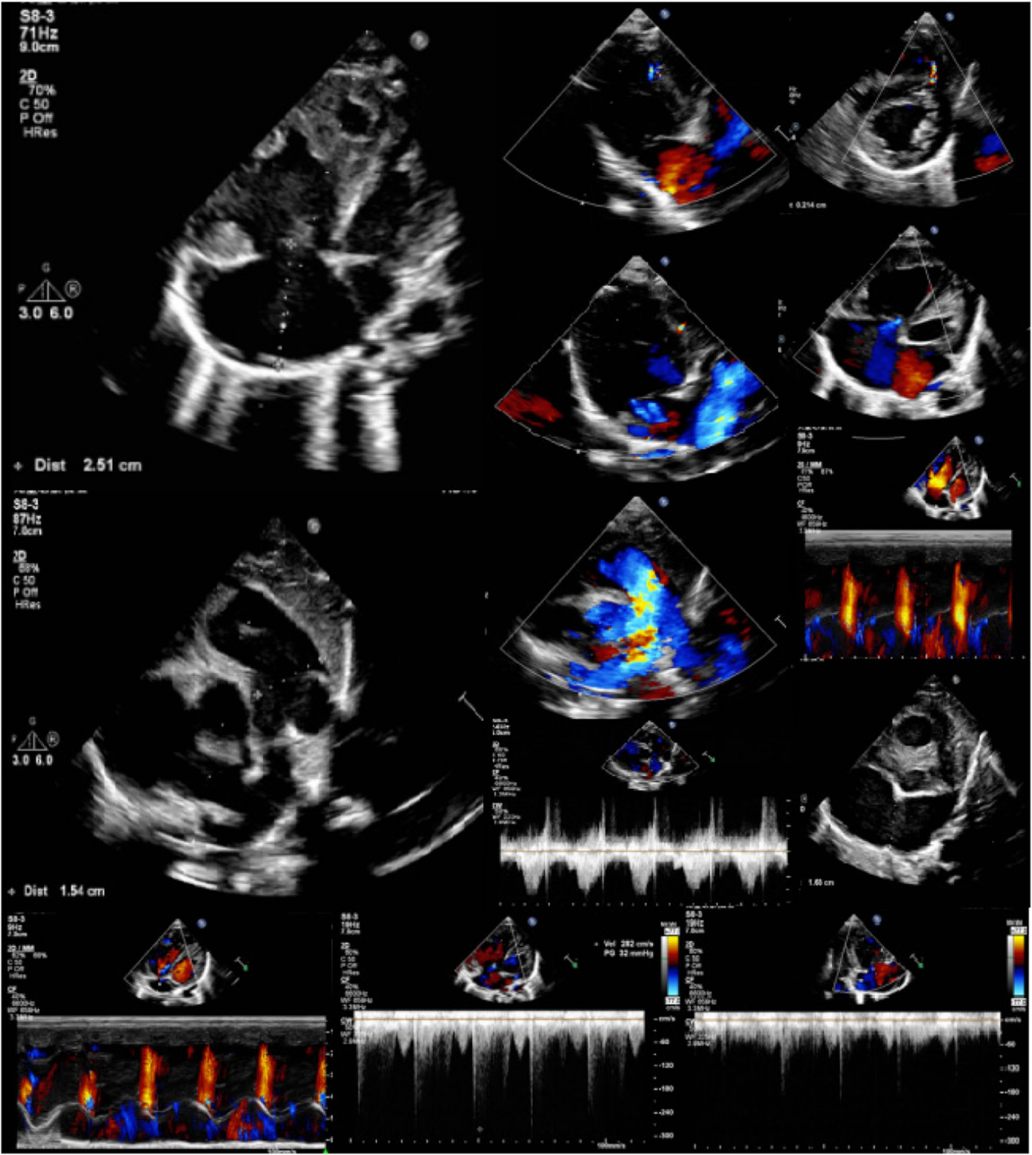
Echocardiography showed atrial septal defect, ventricular septal defect, tricuspid regurgitation (mild to moderate), mitral regurgitation (mild), pulmonary hypertension, biventricular hypertrophy

A 3-month-old female infant was admitted to the pediatric cardiovascular department at the West China Second University Hospital in 2021 for treatment associated with an unknown cardiac disease, and then referred to the medical genetics department for genetic diagnosis. The patient was 60 cm in height and 5 kg in weight; her head circumference was 41 cm, normal for her age. She was also in a good mental state and eating well. She was born full term to a 30-year-old woman who had a previous spontaneous abortion at 24 gestational weeks because of gestational hypertension and placenta abruption during vaginal delivery. There was no apparent abnormality in the appearance of the abortus and there were no remarkable findings during the prenatal examinations (no medical records were provided) for this child. A cardiac murmur was identified three days after birth, and an atrial septal defect and ventricular septal defect were detected by color Doppler ultrasound. In addition, patient was found to have 2–3 toe syndactyly on their left foot at day 4 post birth (Fig. 1**)**. Horizontal nystagmus was found in both eyes at the age of 2 + months, lasting for several minutes per episode with an irregular pattern of presentation. Ultrasound showed that there were punctate echoes in the anterior part of the vitreous dark area in both eyes. The patient had normal motor development, and there was no obvious abnormality in either her hearing or vision at follow up. The patient was noted to cough occasionally, accompanied by rapid breathing, without fever, cyanosis, vomiting and diarrhea, or disturbance of consciousness. She came to the pediatric department of our hospital for further treatment. Echocardiography in our hospital showed congenital heart disease with a huge atrial septal defect: the defect was about 16 mm * 13 mm * 10 mm(anterior stump was 0 mm, posterior stump was 2 mm, upper stump was 3 mm). Color Doppler showed that flow at the atrial level was bidirectional but mainly from left to right, whereas the flow at the ventricular level indicated a muscular ventricular septal defect. Patient also experienced mild to moderate tricuspid regurgitation, mild mitral regurgitation, and pulmonary hypertension due to the huge atrial septal defect and muscular ventricular septal defect (Fig. 2). Left ventricular systolic function was normal, and the CT showed that the bilateral lateral ventricles were full, and there were no abnormal density shadows in the brain parenchyma. Serum troponin was elevated to 0.234 ug/L(normal range 0–0.06), and the diagnosis for this patient in the pediatric cardiovascular department was as follows: atrial septal defect, ventricular septal defect, tricuspid regurgitation (mild to moderate), mitral regurgitation (mild), pulmonary hypertension, sinus tachycardia, right axis deviation + 180°, Biventricular hypertrophy, left 2–3 toe syndactyly, horizontal nystagmus in both eyes, true microphthalmos in the right eye and congenital cataract in the left eye. Given the confluence of these symptoms, we suspected our patient had OFCD syndrome; she was referred to the medical genetics department for further genetic diagnosis.

**Fig. 3:**
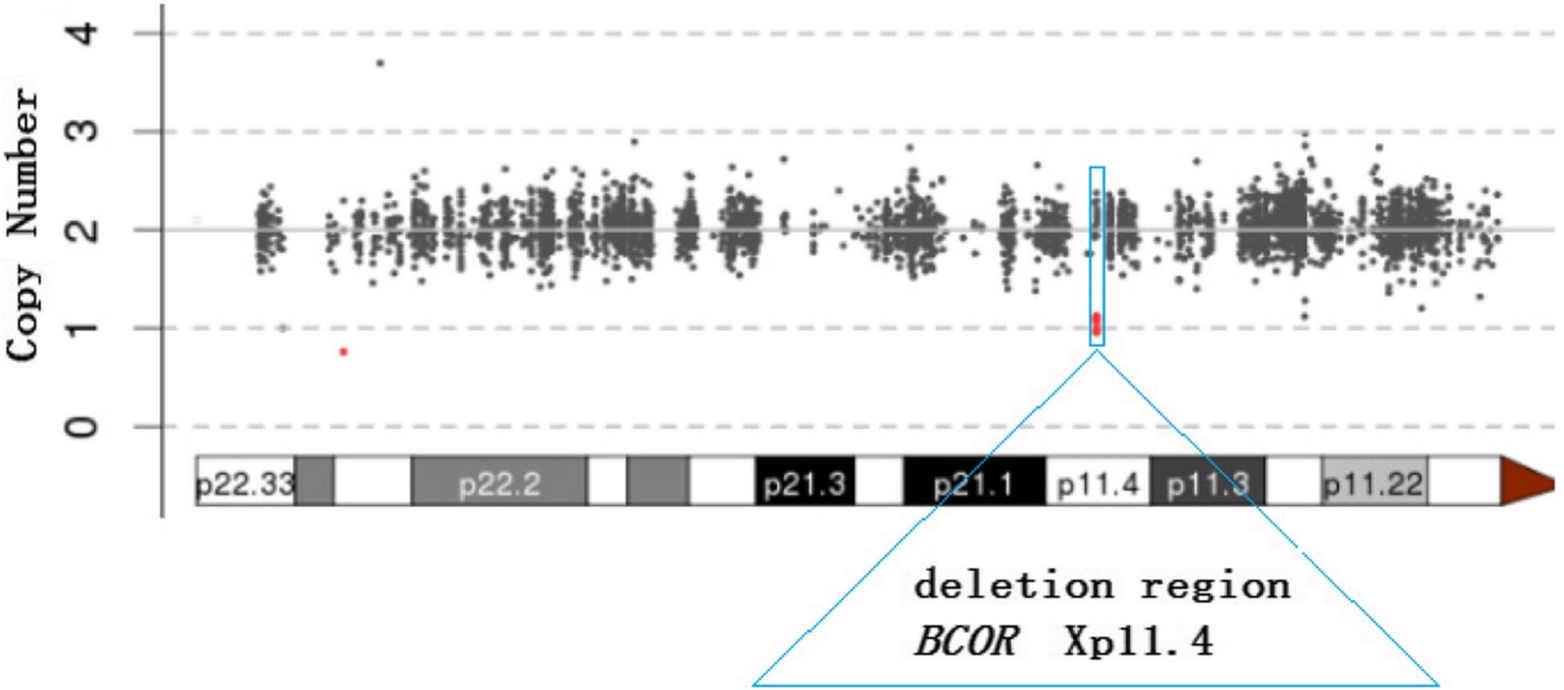
WES(Whole exome sequencing) scatter plot describing the heterozygous loss of Delxp11.4 (about 10.71 KB) in the proband

Whole exon sequencing (WES) produced a scatter diagram that suggests that the proband experienced a mutation, Delxp11.4 (about 10.71 KB), with a copy number of 1 (Fig. 3)**.** Searches of the database failed to identify any single nucleotide variation with definite or probable pathogenicity in these WES sequences.

**Fig. 4:**
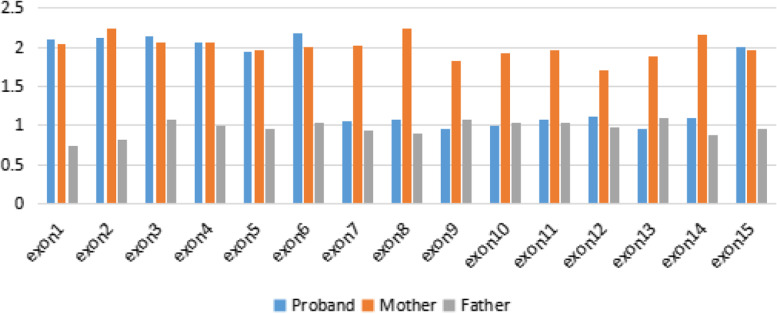
The CSCORE.CNV(Copy number variations) results following WES(Whole exome sequencing): exons 7 to 14 of the *BCOR* gene presented with only a single copy in the proband, and the rest of the *BCOR* gene presented with a copy number of two. Both maternal and paternal data presented with normal copy numbers for the whole gene

CSCORE.CNV evaluation of the WES data showed that the proband presented with a copy number variation for exons 7–14 in the *BCOR* gene, with these exons being present as only a single copy. Exon 1–6 and exon 15 were shown to have a copy number of two. The paternal data revealed that he encoded a single copy of each exon whereas the maternal data presented with two copies of each exon, as expected (Fig. 4).

**Fig. 5:**
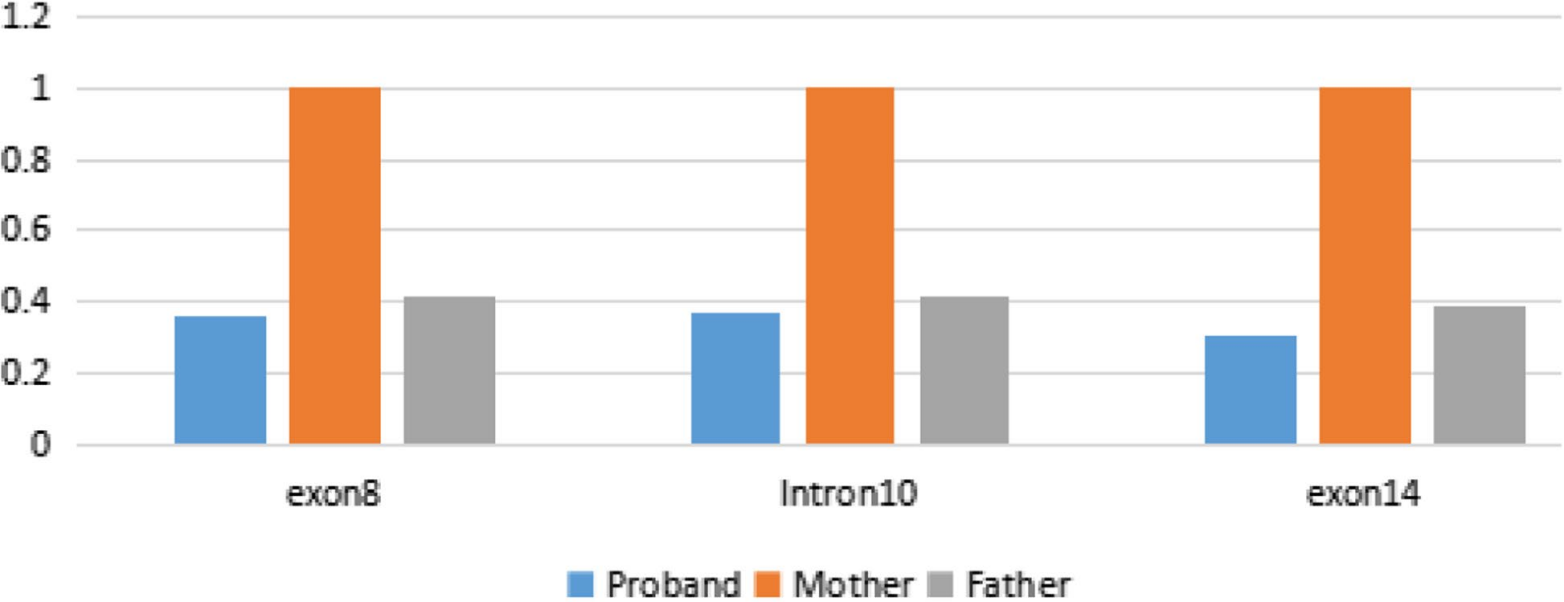
qPCR(quantitative Polymerase Chain Reaction) verification of the WES(Whole exome sequencing) experiments. Both the father and proband presented with half the number of copies for exon 8, intron 10 and exon 14 when compared to the mother

Genomic qPCR revealed that both the father and proband presented with half the number of copies of exon 8, intron 10, and exon 14 found in the mother (Fig. 5).

Taken together these results show that the patient experienced a heterozygous loss of exons 7–14 in their *BCOR* gene whereas both her parents presented with a normal genotype. When we combined this with the clinical phenotype, it was easy to confirm a diagnosis of OFCD syndrome in this patient, likely as a result of the heterozygous deletion of exons 7–14 of the *BCOR* gene.

## Discussion and conclusions

Here we describe the case of a female infant presenting with the typical clinical features of OFCD syndrome. OFCD remains a rare genetic pathological syndrome with no more than 100 cases reported to date [6]. We have summarized the OFCD cases published in recent 5 years (Table 2). According to the summarized phenotypic findings in thosed cases, we found that although OFCD syndrome involves the malformation of multiple organs and systems, including various ocular, facial, cardiac, and dental features, the most characteristic diagnostic symptom for OFCD is teeth abnormality [7]. This means diagnosis in infants, prior to tooth eruption, is difficult. In our case, the patient presented with typical ocular, facial, cardiac, and skeletal abnormalities resulting from a novel mutation in the *BCOR* gene, causing the heterozygous loss of exons 7–14 of this gene. To the best of our knowledge, this mutation has not been previously reported in association with OFCD syndrome. Our patient is also the youngest case of OFCD reported in the recent 5 years, which could be easily misdiagnosed for other diseases such as congenital heart disease because the lack of the typical teeth abnormality during the infant age. Thus, this case expand the OFCD diagnosed age, and should attract more attention from the cardiovascular pediatricians.

**Table 2:** Summary of the OFCD syndrome cases published in recent 5 years

Author	Case	Gender	Age year	Ocular anomalies	Dental anomalies	Facial anomalies	Other anomalies	BCOR mutation
James JO et al. 2017 [8]	1	female	0.58	Microphthalmia, bilateral cataracts	gum hypertrophy	underdevelopment of the midface, supraorbital	Craniosynostosis, ventricular, and atrial septal defects	c.4540C>T
Joji Kato et al. 2018 [9]	2	female	10	congenital cataracts	malocclusion	elongated, with broadening of the nasal tip biprotrusive with a thick lower lip	hammer toe	Exon 4 c.265G>A
Jingshang Zhang et al.,2019 [5]	3	female	4	congenital cataracts	dental caries	flat and slightly long, broad nasal tip with separation of anterior cartilage, protruding ears	bilateral papilloma of choroid plexus, supratentorial hydrocephalus	Exon 4 c. 1296T>A
Nicola Ragge et al. 2019 [10]	4	female	13	congenital cataracts	delayed loss of primary dentition, double row of teeth	no	2nd toe clinodactyly	c.2428C>T
Nicola Ragge et al. 2019[10]	5	male	21	microphthalmia	recurrent dentalinfections	midface hypoplasia, nasal anomalies, ear anomalies	triple heart sounds 5th figure clinodactyly	c.254C>T
Nicola Ragge et al. 2019 [10]	6	female	3	Microphthalmia congenital cataract	late eruption of first, teeth, abnormal crown, canines and incisors	ear anomalies	long finger	c.1209_1210delCC
Nicola Ragge et al. 2019 [10]	7	male	0.75	posterior embryotoxon	posterior embryotoxon	ear anomalies	atrial septal defect, long figure, 4–5 camptodactyly	c.4807A>C
Nicola Ragge et al. 2019 [10]	8	male	5	posterior embryotoxon	posterior embryotoxon	ear anomalies cleft palate	atrial septal defect, camptodactyly, all finger, fetal toe pads	c.4807A>C
Nicola Ragge et al. 2019 [10]	9	female	17	congenital cataract, glaucoma	delayed loss of primary dentition,radiculomegaly	cleft palate, nasal anomalies	5th figure clinodactyly, 2–3 toe syndactyly	c.4700_4718dup
Nicola Ragge et al. 2019 [10]	10	female	15	congenital cataract, glaucoma	delayed loss of primary dentition, radiculomegaly, teeth misaligned	nasal anomalies	atrial septal defect, long figure, long toes	c.867G>A
Nicola Ragge et al. 2019 [10]	11	female	6	congenital cataract	agenesis two lateral incisors	ear anomalies	long figure, long toes	c.2947_2948insTGC ATACT
Nicola Ragge et al. 2019 [10]	12	male	27	anophthalmia	no	Nasal anomalies	2–3 toe syndactyly	c.254C>T
Nicola Ragge et al. 2019 [10]	13	female	9	microphthalmia, congenital cataract	late eruption of first teeth delayed loss of primary dentition	nasal anomalies ear anomalies cleft palate	long figure, long toes	c.3153G>A
Nicola Ragge et al. 2019 [10]	14	female	11	congenital cataract	late eruption of first teeth delayed loss of primary dentition	nasal anomalies ear anomalies	2-3 syndactyly 2nd toe clinodactyly, 4^th^ toe camptodactyly	c.4850T>G
Nicola Ragge et al. 2019 [10]	15	male	3	posterior embryotoxon	no	no	no	c.4741 + 1G>A
Nicola Ragge et al. 2019 [10]	16	female	2	microphthalmia, congenital cataract	late eruption of first teeth	no	long figure short metacarpals	c.4402C>T
Nicola Ragge et al. 2019 [10]	17	female	3	congenital cataract	late eruption of first teeth	nasal anomalies, cleft palate	no	c.4601_4602insCT
Nicola Ragge et al. 2019 [10]	18	female	14	congenital cataract glaucoma	no	nasal anomalies, ear anomalies, cleft palate	long toes, increased sandal gap	c.3116_3117dup
José Martinho et al. 2019 [11]	19	female	26	cataracts	dentition with numerous missing teeth	a long, narrow face with a bifid tip of the nose	prolapsed mitral valve, a misalignment of her thumbs, valgus foot condition	unknown
Song Hee Oh et al. 2019 [12]	20	female	31	a cloudy lens with symptoms similar to cataract and visual impairment.	radiculomegaly of all quadrants of the jaws	elongated face, facial asymmetry	a blunt gonial angle	unknown
T.M.Morgan et al. 2019 [13]	21	female	15	congenital cataracts	persistent baby teeth, , delayed eruption of secondary teeth, and long roots of her teeth	flat feet high-arched palate facial features	Clinodactyly ventriculoseptal defect	exon 4 c.776C>A
T.M.Morgan et al. 2019 [13]	22	female	10	Congenital cataracts glaucoma microphthalmos	long roots of her teeth with one missing tooth and first primary tooth loss at 6-7 years of age	no	atrial septal defect aberrant right subclavian artery	c.2514del(G)

*BCOR* mutations are present in almost all reported cases of OFCD syndrome, which suggests that these mutations are the primary cause of this pathology. The *BCOR* gene is located on the X chromosome, in the Xp11.4 locus, and derives its name from its function as an interacting corepressor of BCL-6 that enhances BCL-6-mediated transcriptional repression [4]. *BCOR* is made up of 15 exons, encoding three homologous isomers, which produce 1,721 amino acid peptides (NP_001116855.1) [5]. *BCOR* plays a central role in maintaining pluripotency, inducing differentiation and determining cell fate [4], and the BCOR protein is widely expressed in various human tissues. This means that BCOR is of clinical interest for its important roles in development [6].

Distinct classes of *BCOR* mutations result in two different rare X-linked syndromes: OFCD syndrome and MAA2 associated Lenz microphthalmia [1]. Lenz microphthalmia is inherited in an X-linked recessive pattern and is characterized by microphthalmia, mental retardation, and skeletal and other anomalies, while OFCD is inherited in an X-linked dominant pattern with presumed male lethality [1]. OFCD patients can have defects in the craniofacial, skeletal, and cardiovascular systems, and all are heterozygous for a mutant *BCOR* allele [2, 10, 14, 15]. All the reported OFCD mutations, including nonsense, frameshift insertions/deletions/duplications, splicing mutations, and large deletions, are predicted to cause OFCD clinical manifestations via gene loss or protein truncation and/or nonsense-mediated decay interrupting BCOR function [2, 7, 10, 15–19]. The severity of the OFCD phenotype varies widely because of the individual variation in the proportion of key tissue cells that have a transcriptionally active X chromosome with the *BCOR* mutation [2, 10, 15]. This was proven to be true by the X-inactivation analyses of leukocytes from OFCD patients, which showed 96–100% allelic skewing helped cells expressing the wild type allele of *BCOR* [1, 20].

Female OFCD patients may present with short stature and low weight or growth retardation and commonly display a distinctive craniofacial phenotype, including microcephaly, long narrow facial features, long philtrum, microcornea, congenital cataracts, microphthalmia, clinical anophthalmia, vision loss, secondary glaucoma with a high nasal bridge, or bifid nasal tip. The dental abnormalities linked to OFCD include canine radiculomegaly, delayed dentition, persistent primary teeth, oligodontia, malocclusion, supernumerary teeth. In addition, cleft palate, submucous cleft palate and Bifid uvula occur in over a quarter of patients. Patients may also have abnormalities in their ear structures, including large anteverted ears, asymmetric ears, or posteriorly rotated ears, and 9% of patients have mild conductive or sensorineural hearing loss. OFCD skeletal defects include skull defects (microcephaly), spine abnormalities (scoliosis), limb defects (flexion contractures, limited supination, radioulnar synostosis), and feet defects (hammer toe, toe syndactyly, and radioulnar synostosis) [1, 2, 10, 15, 21]. Cardiovascular defects are also common in OFCD patients with up to 67% of OFCD patients presenting with at least one cardiovascular defect. Amongst these, septal defects (atrial, ventricular, or both) are the most common and are found in 50% of the OFCD patients examined [10, 15].

The treatment plan for OFCD patients involves a multidisciplinary approach to treat the cardiac, skeletal, facial, and dental disharmony in these patients, and early diagnosis and treatment is crucial for patient prognosis. Our patient was diagnosed early enough to allow for the cardiac defects to be treated first, and she will be closely followed up to ensure that any new presentations are treated in a timeous manner. Meanwhile, due to the involvement in the tumorigenesis of the *BCOR* gene, the patient should also be closely followed up for any signs of cancer. The mutation in the *BCOR* of this patient is novel, and her parents were not affected, suggesting that this was a de novo mutation or inherited from a parent who was a Gonad mosaic for this kind of mutation. Given this, we suggest that the reproductive plan for these parents is to include prenatal diagnosis targeting the *BCOR* gene for any additional pregnancies in case that any of the parents was a Gonad mosaic for this kind of mutation.

In summary, we report the case of a 3-month-old female OFCD patient with typical clinical phenotype, including cardiac defects, toe syndactyly, true microphthalmos, and congenital cataracts, presenting with a novel heterozygous deletion of exons 7–14 of the *BCOR* gene. Her parents were not affected, and to the best of our knowledge, she is the youngest OFCD patient ever reported, which is vital for her prognosis. Given the clinical dependance on dental deformities to diagnose OFCD, our ability to diagnose an infant with OFCD, prior to tooth eruption, should help to establish a clinical diagnosis of this syndrome in the absence of tooth involvement.

## Data Availability

The raw datasets used and analysed during the current study are not deposited in publicly available repositories because of considerations about the security of human.

## Abbreviations

OFCD: Oculo-Facio-Cardio-Dental syndrome
*BCOR BCL6*: Corepressor gene
*DNA*: Deoxyribonucleic acid
WES: Whole exome sequencing
CNV: Copy number variations
SNP: Single Nucleotide Polymorphism
qPCR: Quantitative Polymerase Chain Reaction
CT: Computed Tomography
GATK: Genome Analysis ToolKit
MAA2: Microphthalmia with Associated Anomalies 2
XHMM: eXome Hidden Markov Model

## References

[1] Ng D, Thakker N, Corcoran CM, Donnai D, Perveen R, Schneider A (2004). Oculofaciocardiodental and Lenz microphthalmia syndromes result from distinct classes of mutations in *BCOR*. Nat Genet.

[2] Feberwee HE, Feenstra I, Oberoi S, Sama IE, Ockeloen CW, Clum F (2014). Novel *BCOR* mutations in patients with oculofaciocardiodental (OFCD) syndrome. Clin Genet.

[3] Hayward JR (1980). Cuspid gigantism. Oral Surg Oral Med Oral Pathol.

[4] Huynh KD, Fischle W, Verdin E, Bardwell VJ (2000). *BCOR*, a novel corepressor involved in BCL-6 repression. Genes Dev.

[5] Zhang J, Jia H, Wang J, Xiong Y, Li J, Li X (2019). A novel deletion mutation, c.1296delT in the *BCOR* gene, is associated with oculo-facio-cardio-dental syndrome C 1296delt. Sci China Life Sci.

[6] Hamline MY, Corcoran CM, Wamstad JA, Miletich I, Feng J, Lohr JL (2020). OFCD syndrome and extraembryonic defects are revealed by conditional mutation of the Polycomb-group repressive complex 1.1 (PRC1.1) gene *BCOR*. Dev Biol.

[7] Surapornsawasd T, Ogawa T, Tsuji M, Moriyama K (2014). Oculofaciocardiodental syndrome: Novel *BCOR* mutations and expression in dental cells. J Hum Genet.

[8] James JO, Eoghan L, Dylan JM, William R (2017). Oculo–Facio–Cardio–Dental Syndrome with Craniosynostosis, Temporal Hypertrichosis, and Deafness. Am J Med Genet A.

[9] Kato JJ, Kazuhiko K, Fumikazu K (2018). New Radiological Findings and Radiculomegaly in Oculofaciocardiodental Syndrome with a Novel BCOR Mutation A Case Report. Medicine (United States).

[10] Ragge N, Isidor B, Bitoun P, Odent S, Giurgea I, Cogné B (2019). Expanding the phenotype of the X-linked *BCOR* microphthalmia syndromes. Hum Genet.

[11] Martinho J, Hugo F, Siri P, Anabela P, Carlos MM, Eunice C, Manuel MF (2019). Oculo-Facio-Cardio-Dental Syndrome: A Case Report about a Rare Pathological Condition. INT J ENV RES PUB HE.

[12] Oh SH, Ju HK, Yu KS, Sae RL, Yong SC, Eui HH (2019). Radiculomegaly of Canines in Oculofaciocardiodental Syndrome. ORAL RADIOL.

[13] Morgan TM, Colazo JM, Duncan L, Hamid R, Joos KM (2019). Two Cases of Oculofaciocardiodental (OFCD) Syndrome Due to X-Linked BCOR Mutations Presenting with Infantile Hemangiomas: Phenotypic Overlap with PHACE Syndrome. Case Rep Genet.

[14] Narumi S (2018). Rare monogenic causes of primary adrenal insufficiency. Curr Opin Endocrinol Diabetes Obes.

[15] Hilton E, Johnston J, Whalen S, Okamoto N, Hatsukawa Y, Nishio J (2009). *BCOR* analysis in patients with OFCD and Lenz microphthalmia syndromes, mental retardation with ocular anomalies, and cardiac laterality defects. Eur J Hum Genet.

[16] Danda S, Van Rahden VA, John D, Paul P, Raju R, Koshy S (2014). Evidence of germline mosaicism for a novel *BCOR* mutation in two Indian sisters with oculo-facio-cardio-dental syndrome. Mol Syndromol.

[17] Di Stefano C, Lombardo B, Fabbricatore C, Munno C, Caliendo I, Gallo F (2015). Oculo-facio-cardio-dental (OFCD) syndrome: The first Italian case of *BCOR* and co-occurring *OTC* gene deletion. Gene.

[18] Ma AS, Grigg JR, Ho G, Prokudin I, Farnsworth E, Holman K (2016). Sporadic and familial congenital cataracts: Mutational spectrum and new diagnoses using next-generation sequencing. Hum Mutat.

[19] Zhou Y, Wojcik A, Sanders VR, Rahmani B, Kurup SP (2018). Ocular findings in a patient with oculofaciocardiodental (OFCD) syndrome and a novel *BCOR* pathogenic variant. Int Ophthalmol.

[20] Hedera P, Gorski JL (2003). Oculo-facio-cardio-dental syndrome: Skewed X chromosome inactivation in mother and daughter suggest X-linked dominant inheritance. Am J Med Genet A.

[21] Suzumori N, Kaname T, Muramatsu Y, Yanagi K, Kumagai K, Mizuno S (2013). Prenatal diagnosis of X-linked recessive Lenz microphthalmia syndrome. J Obstet Gynaecol Res.

